# Supplementary figures and images for: Heparan sulfate-dependent phase separation of CCL5 and its chemotactic activity
Source: eLife. 2024 Jul 1;13:RP93871. doi: 10.7554/eLife.93871 (PMC11216747; doi:10.7554/eLife.93871)

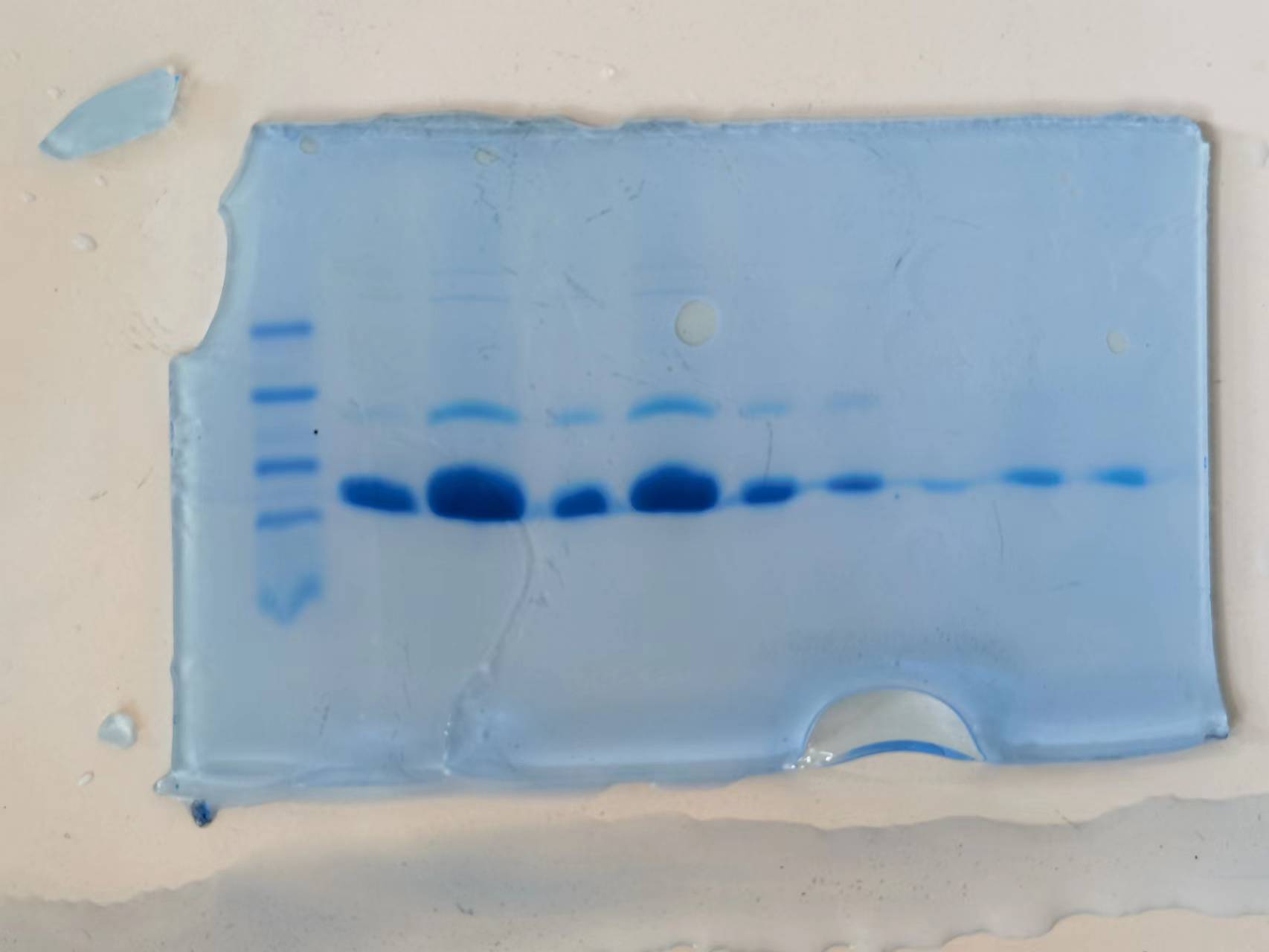

Supplement: Figure 1—figure supplement 1—source data 1. [file elife-93871-fig1-figsupp1-data1.zip › Figure1-figure supplement 1-source data 1.jpg]

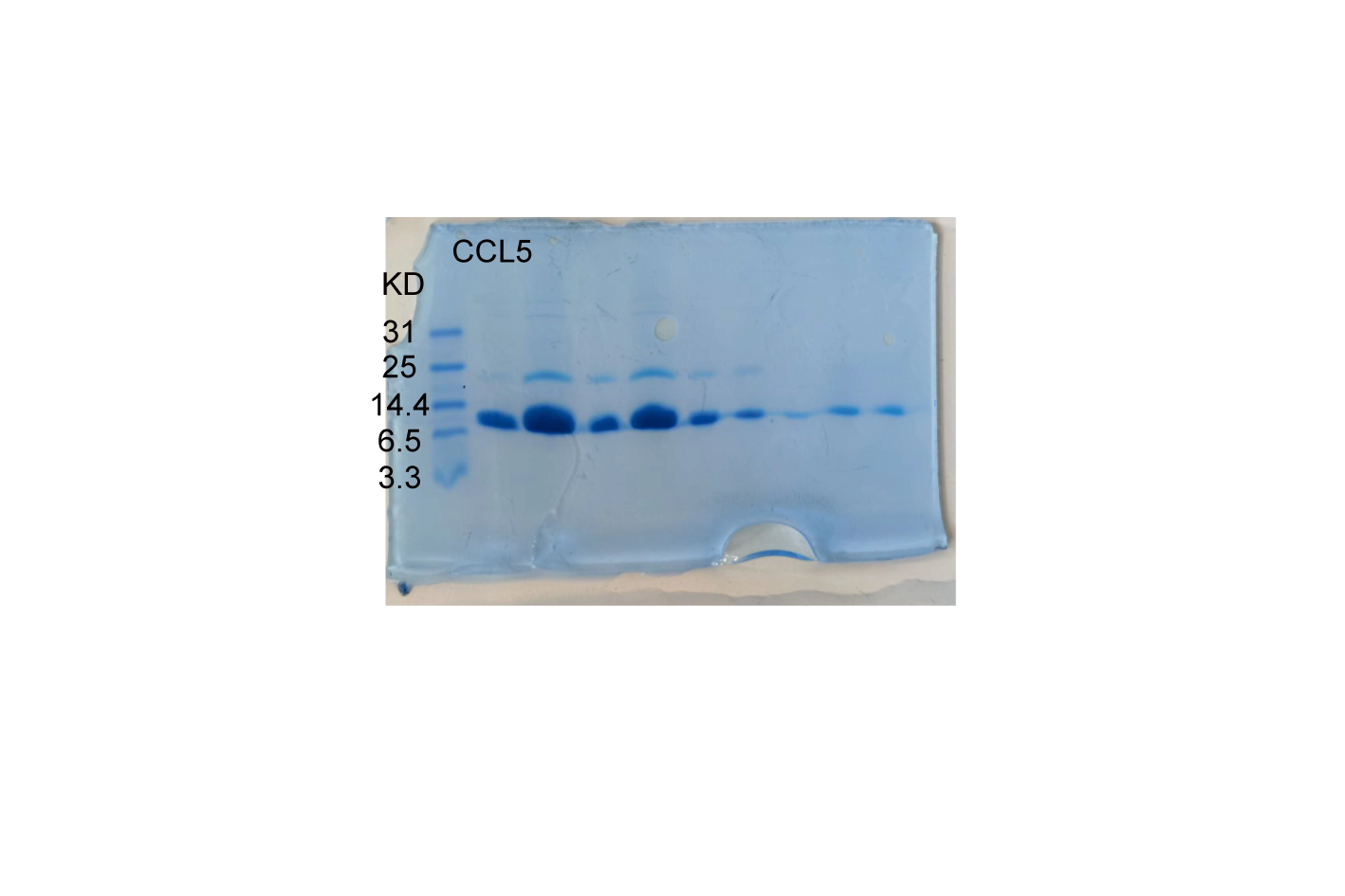

Supplement: Figure 1—figure supplement 1—source data 2. [file elife-93871-fig1-figsupp1-data2.zip › Figure1-figure supplement 1-source data 2.tif]

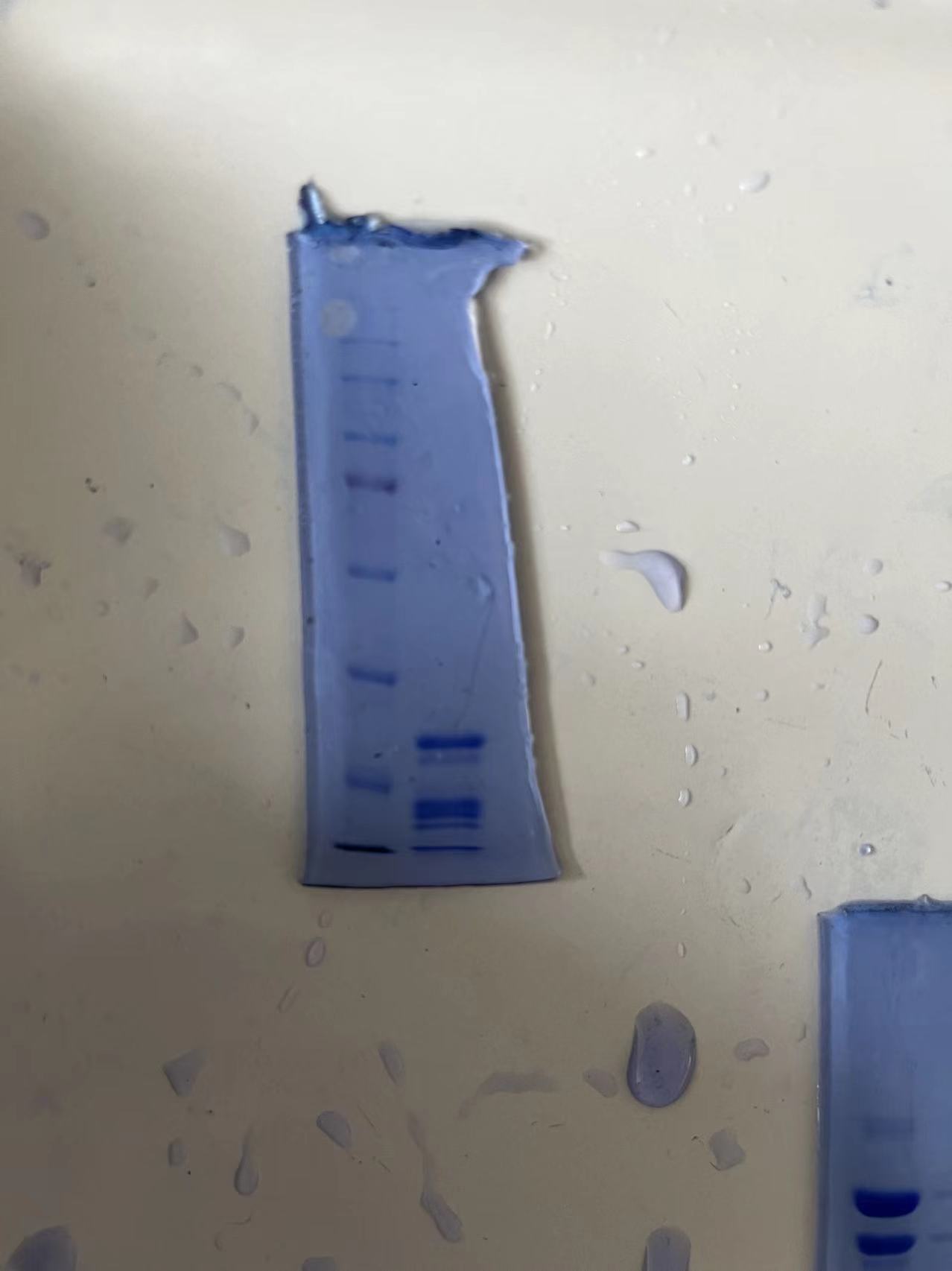

Supplement: Figure 1—figure supplement 1—source data 3. [file elife-93871-fig1-figsupp1-data3.zip › Figure1-figure supplement 1-source data 3.jpg]

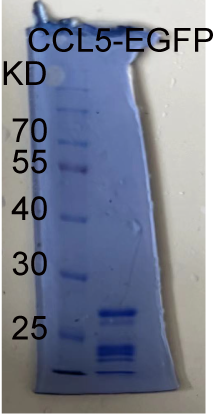

Supplement: Figure 1—figure supplement 1—source data 4. [file elife-93871-fig1-figsupp1-data4.zip › Figure1-figure supplement 1-source data 4.tif]
